# Genetically Engineered Proteins to Improve Biomass Conversion: New Advances and Challenges for Tailoring Biocatalysts

**DOI:** 10.3390/molecules24162879

**Published:** 2019-08-08

**Authors:** Lucas Ferreira Ribeiro, Vanesa Amarelle, Luana de Fátima Alves, Guilherme Marcelino Viana de Siqueira, Gabriel Lencioni Lovate, Tiago Cabral Borelli, María-Eugenia Guazzaroni

**Affiliations:** 1 Department of Biology, Faculdade de Filosofia, Ciências e Letras de Ribeirão Preto, University of São Paulo, Ribeirão Preto 14040-901, Brazil; 2 Department of Microbial Biochemistry and Genomics, Biological Research Institute Clemente Estable, Montevideo, PC 11600, Uruguay; 3 Department of Biochemistry and Immunology, Faculdade de Medicina de Ribeirão Preto, University of São Paulo, Ribeirão Preto 14049-900, Brazil

**Keywords:** mutagenesis, recombination, directed evolution, semi-rational design, screening or selection, computational design

## Abstract

Protein engineering emerged as a powerful approach to generate more robust and efficient biocatalysts for bio-based economy applications, an alternative to ecologically toxic chemistries that rely on petroleum. On the quest for environmentally friendly technologies, sustainable and low-cost resources such as lignocellulosic plant-derived biomass are being used for the production of biofuels and fine chemicals. Since most of the enzymes used in the biorefinery industry act in suboptimal conditions, modification of their catalytic properties through protein rational design and *in vitro* evolution techniques allows the improvement of enzymatic parameters such as specificity, activity, efficiency, secretability, and stability, leading to better yields in the production lines. This review focuses on the current application of protein engineering techniques for improving the catalytic performance of enzymes used to break down lignocellulosic polymers. We discuss the use of both classical and modern methods reported in the literature in the last five years that allowed the boosting of biocatalysts for biomass degradation.

## 1. Introduction 

Lignocellulosic biomass derived from plant cell wall is a very abundant (global production of ~200 billion tons/year [1]), low-priced, and environmentally friendly raw material that can be used in a wide range of applications, from biofuels and biomaterials to value-added chemical production [2,3,4]. Lignocellulose is a complex matrix composed of carbohydrate polymers (cellulose and hemicellulose) and phenolic polymers (lignin). The enzymatic degradation of these polymers is accomplished by the concerted action of multiple enzymes, which leads to simple sugars that can be fermented to ethanol and other products of high relevance in the industry [3]. Nevertheless, its crystal structure makes lignocellulosic material recalcitrant and resistant to biological degradation, which motivated scientists to pursue novel enzymes with enhanced kinetic parameters and to modify previously described enzymes, to improve the biomass saccharification process [5,6].

Most of the enzymes used in industry—identified from cultured microorganisms or metagenomic approaches—need to work cost-effectively within stipulated manufacturing parameters [7]. However, enzymes commonly used in industry (including biomass-degrading enzymes) are not working in optimized conditions, and industrial processes have to be adjusted to adapt to these suboptimal biocatalysts [8]. In this context, one of the main alternatives to revert this situation is to chemically or genetically manipulate proteins to improve their properties. The biocatalysis field experienced tremendous expansion over the past decades and can be summarized in three specific waves of development [9]. During the first and second waves, enzyme immobilization was the foremost example of chemical manipulation, and it is still a useful tool to improve protein features. Overall, in this approach, an enzyme is confined, through molecular forces, into a matrix or a nanoparticle (enzyme nanoparticles) to generate a reusable, stable, and highly active catalytic species. Chemical-based approaches are already successfully used to improve biomass conversion [10,11,12]. 

**Figure 1:**
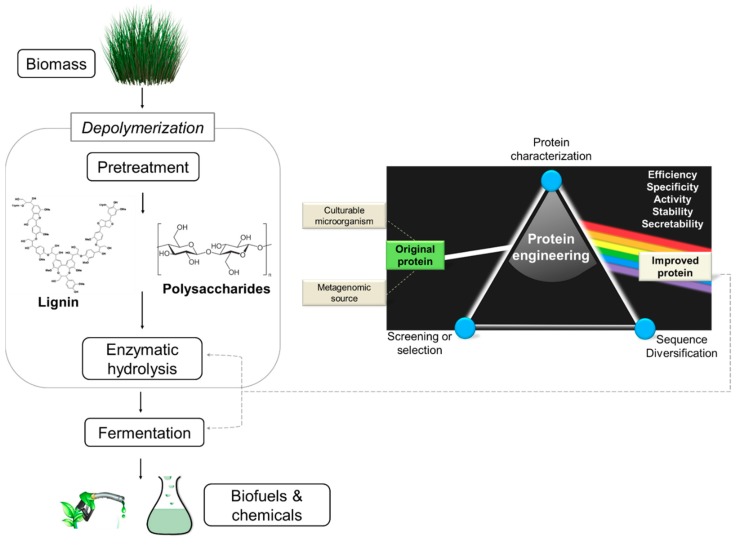
General schema used for the development of an ideal biocatalyst with potential application in bio-based economy procedures, such as plant-derived biomass degradation for biofuels or fine chemical production.

On the other hand, the third and most recent wave started with the development of technologies to engineer an enzyme genetically (termed hereafter simply as “protein engineering”). The engineering can be performed through two main approaches, comprising directed evolution and rational design [13,14]. Directed evolution simulates, in a faster way, the natural selection process. In this approach, the protein coding sequence is altered—via creation of mutations, insertions, deletions, recombination, duplications, etc.—generating a group of different proteins; then, these variants are screened for one or more improved traits [15]. Rational design uses prior knowledge on protein structure and computational tools in order to deliberately design novel proteins [16]. Both protein engineering approaches—directed evolution and rational design—are applied to improve protein properties of the enzymes involved in depolymerization and fermentation of the lignocellulosic material to produce biofuels or fine chemicals (Figure 1).

This review focuses on the latest advances in genetically protein engineering approaches aiming to achieve ideal candidate enzymes with particular features for biomass conversion, covering some of the most recent and relevant findings in this topic. As the research field is pervasive, our primary focus is to direct readers to meaningful references where details about the progress made in this specific area can be found, with emphasis on studies done in the last five years. We also address the use of both classical and innovative methods—such as synthetic biology approaches, engineering of components related to vesicle trafficking, and microfluidic-based deep mutational scanning—for maximizing the efficiencies of biocatalysts used to break down lignocellulosic polymers. 

## 2. Protein Engineering 

### 2.1. General Tools 

#### 2.1.1. Directed Evolution

**Figure 2:**
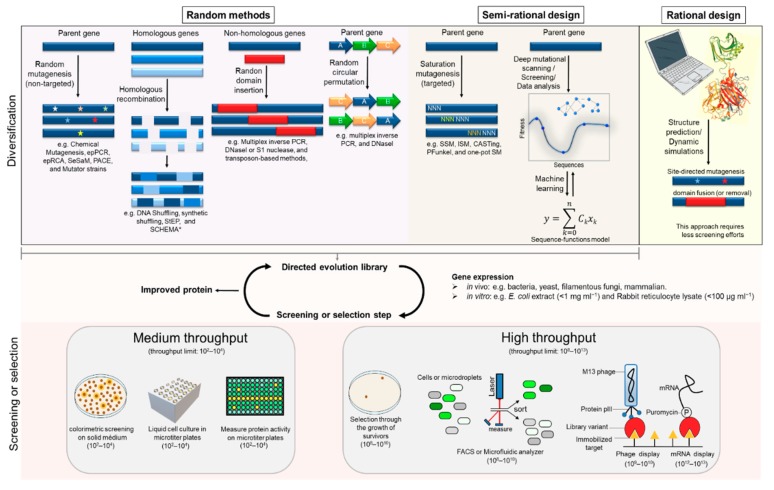
Overview of protein engineering strategies. Protein properties can be improved by using directed evolution (random modes or semi-rational design) or rational design approaches through a process of genetic diversification, gene expression, and screening or selection. For directed evolution, protein variants can be optimized using iterative cycles of this process. ^*^ SCHEMA uses structural information to perform recombination; thus, this method can be classified as a semi-rational approach.

Protein engineering is a field in continuous growth, in which new tools for protein tailoring are continuously developed. Many of these tools are focused on evolving proteins through directed evolution—an approach that was awarded the Nobel prize in chemistry in 2018 [17,18]. This powerful approach mimics the natural evolution process in a short timescale and implies little or no knowledge about the tertiary structure of the protein. Starting from a parental protein, a library of genetically modified variants is generated. Subsequently, proteins with improved traits are identified by screening or selection methods. These variants can be further optimized through successive cycles of diversification, screening, or selection. Since a complete analysis of the protein sequence space is impossible, due to its astronomical size, diversification and screening methods should be carefully chosen. A multitude of techniques to perform directed evolution experiments, either *in vivo* or *in vitro*, were developed in the last decades. Figure 2 briefly summarizes the most common tools for gene diversification and screening or selection. For a more comprehensive description of relevant protein engineering methodologies, detailed book chapters and review articles may be consulted [19,20,21,22,23].

Diversification can be achieved through non-recombinant or recombinant methodologies. These methods can be divided into two approaches, random or semi-rational. The most common method to generate random mutagenesis is error-prone PCR (epPCR) [24,25]. However, other (non-physical/chemical) methods also became more popular such as error-prone rolling circle amplification (epRCA) [26], sequence saturation mutagenesis (SeSaM) [27], and mutator strains (e.g., phage-assisted continuous evolution (PACE) [28] or XL1-red *Escherichia coli* [29]). 

In addition to the mutagenesis methods, genetic recombination can be used to improve protein fitness. Recombination methods allow clusterization of desirable features from either homologous or non-homologous genes. Well-established techniques for random homologous recombination include DNA shuffling [30], synthetic shuffling [31], and staggered extension process (StEP) [32]. In addition, homologous recombination can be performed using a semi-rational approach known as SCHEMA [33]. This method uses three-dimensional structures and a computational algorithm to guide the recombination. The homologous recombination methods described above are frequently used after rounds of mutagenesis, to combine mutations from distinct variants. However, in some situations, it would be interesting to recombine genes or DNA fragments with low or no homology. Random non-homologous recombination can be useful, for instance, to combine two or more different domains to generate multifunctional chimeric proteins [34,35,36]. Methods using multiplex inverse PCR, DNaseI or S1 nuclease, and transposon are among the most common techniques to create random domain insertion in a homology-independent way [23,34,37,38,39]. 

Another method for diversification is known as random circular permutation [23,40,41]. In this method, the intramolecular order of amino acids is rearranged through linking the original N- and C-termini of the protein and opening the gene in a new point to yield new N- and C-termini. These new N- and C-termini can be created randomly by multiplex inverse PCR or DNaseI [23]. Relocation of the N- and C-termini might relax the structural constraints and increase protein flexibility.

On the other hand, in the semi-rational approach used for directed evolution experiments, diversification is focused on “hotspot” residues/regions of the protein. These hotspots can be identified from structural/functional information or from previous random directed evolution experiments. The focus on functionally significant sites generally avoids the screening of large libraries. A focused library can be generated through the replacement of the individual hotspot residues with all possible amino acids by saturation mutagenesis methods like site-saturation mutagenesis (SSM) [42], iterative saturation mutagenesis (ISM) [43], combinatorial active-site saturation test (CAST) [44], PFunkel [45], and one-pot saturation mutagenesis (one-pot SM) [46]. Several saturation mutagenesis methods allow simultaneous codon replacement within multiple targeted sites, generating more extensive libraries. Single or simultaneous saturation mutagenesis use oligonucleotides containing a degenerate codon (e.g., NNN; N = A, T, C, or G) corresponding to the targeted position. The degenerate codons NNN yield all 64 possible codons. However, this codon produces more extensive libraries with an unbalanced amino-acid distribution. For instance, among the 64 possible codons, there are six-fold more codons for serine than for tryptophan or methionine. Thus, codon optimization may be essential to create smaller, so-called smart libraries, as shown in Table 1. 

Due to the large size of the protein sequence space, it is vanishingly rare to find highly functional sequences. Several computational tools were developed to make directed evolution a more efficient process [41,49]. The SCHEMA algorithm is a successful example of how homologous proteins can be recombined with the help of these tools [50]. Usually, the information from unimproved sequences generated by directed evolution experiments is discarded. However, machine-learning methods were recently developed to use this information to optimize the evolution process [51]. The data from directed evolution experiments and DNA deep sequencing analyses can be used to build a “sequence–function model” via machine-learning methods. Therefore, this computational model allows creating and screening combinations of sequence variants with a higher probability of improved function.

#### 2.1.2. Rational Design 

Rational design requires more profound knowledge about the structural or biochemical aspects of the protein. Thus, *a priori* information is used to make specific changes in the protein to achieve the desired improvement on its function [16,52]. One of the most popular methods for rational diversification is site-directed mutagenesis (SDM) [53], which is used to evaluate the effect of one or more amino acids predicted to affect a specific protein feature. Computational tools, such as structure homology modeling and molecular dynamics simulations, can help to predict the importance of residues and suggest putative sites to modify [49]. These tools can also help to choose a specific region to “open up” a protein and insert a new domain (domain insertion) or even to delete a non-essential domain (domains removal). 

Therefore, the appropriate strategy for protein engineering can be chosen based on the feasibility of experimental techniques, availability of the computer algorithms, and the knowledge of protein structure–function. In the next paragraphs, we present several examples of directed evolution and rational design that sought to improve the adaptability of the proteins involved in the biomass conversion process.

### 2.2. Strategies to Improve Biomass Conversion 

#### 2.2.1. Engineering Protein Activity 

Engineering protein activity is one of the main strategies used to improve the performance of lignocellulose degrading enzymes. For the improvement of enzymatic activity through protein engineering, some targets are considered as most effective, such as the modification of catalytic domains by mutagenesis, coupling non-catalytic modules as the carbohydrate-binding modules (CBMs), construction of multi-functional enzymes, and designing cellulosomes. Recent progress for improving activity in biomass-converting enzymes by protein engineering is summarized in Table 2.

Carbohydrate-binding modules (CBMs) are amino-acid sequences capable of binding carbohydrates. CMBs play an important role in directing carbohydrate degrading enzymes toward their substrate [80]; therefore, constructing chimeric proteins fusing CBMs and catalytic domains from a broad range of enzymes is a strategy widely used to improve the conversion of complex macromolecular structures, such as plant cell-wall polymers into mono- or oligomeric molecules. For instance, Duan et al. constructed chimeras by fusing a metagenomic cellulase GH9 (Umce19A) to six CBMs from different families (CBM1, CBM2, CBM3, CBM4, CBM10, and CBM72), with each CBM containing the natural linker from the native enzymes. Catalytic activity and catalytic efficiency (k_cat_/K_M_) of the chimeric CBM-carrying enzymes were higher when compared with the wild-type enzyme. CBM4-Umcel9A exhibited 4.2-, 3.0-, 2.4-, and 6.6-fold enhanced activity against phosphoric acid-swollen cellulose, alkali-pretreated sugarcane bagasse, filter paper powder, and Avicel, respectively, and 4.4-fold enhanced catalytic efficiency when compared with Umcel9A [81]. In another study, Walker et al. evaluated the ability of CBMs from 18 families found in *Ruminoclostridium thermocellum* to modulate the function of the multifunctional GH5 CelE, also from *R. thermocellum*. Chimeras showed up to four-fold enhancement, in both rate and yield, in the hydrolysis of different polysaccharide substrates (cellulose, lichenan, xylan, and mannan) and also with ionic liquid-pretreated switchgrass [82]. Fonseca-Maldonado et al. evaluated the effect of different CBM on *Bacillus subtilis* endoglucanase (BsCel5A) activity by exchanging its inherent CBM3 for the CBM11 from *R. thermocellum* CelH (RtCBM11) which displays broader glucan affinity. BsCel5A-RtCBM11 resulted in a 2.1-fold increase in catalytic efficiency towards β-glucan. [83].

Additionally, engineering CBMs showed attractive gains in the field [84]. Strobel et al. engineered the CBM and linker of *Trichoderma reesei* Cel7A—which is considerably inhibited by lignin—in order to increase the binding specificity for cellulose. By constructing a library varying seven residues in the CBM domain and removing predicted glycosylation sites in the linker, they generated a mutant with 2.5-fold less lignin affinity, which was able to enhance by 40% the rate of glucose released from diluted acid-pretreated *Miscanthus* biomass [85]. In another approach, Furtado et al. created a random mutant library for CBM from *R. thermocellum* and screened for xyloglucan binding, identifying CBM mutants with increased xyloglucan affinity. Based on the affinity characteristics of the screening, they created a CBM quadruple mutant by site-directed mutagenesis [86]. Fusion of GH12 xyloglucanase from *Aspergillus niveus* with mutant CBM showed 38% enhancement in catalytic efficiency when compared to the wild-type CBM GH12 chimera [86]. Similarly, Gunnarsson et al. constructed a combinatorial library making substitutions of 12 residues around the binding site of CBM4-2 from *Rhodothermus marinus* xylanase Xyn10A, which is capable of binding to different xylans and β-glucans. Library construction was followed by a selection procedure using different target substrates, in which some variants showed affinity toward specific carbohydrate polymers, including birchwood xylan, Avicel, and ivory nut mannan, as well as the human glycoprotein immunoglobulin G4 (IgG4) [84]. Therefore, engineered CBMs could serve as a scaffold for evolving new binding specificities.

##### Engineering Cellulosomes and Protein Scaffolds to Improve Biomass Deconstruction

A select number of cellulolytic bacteria can degrade lignocellulosic biomass through nanomachines termed cellulosomes [87]. These multi-protein complexes comprise a non-catalytic scaffoldin which interacts with various enzymes subunits with the help of cohesin and dockerin modules [87]. The cellulosome architecture allows spatial proximity of enzymes which present complementary catalytic activities, enabling synergistic activities, minimizing negative feedback by enzymatic products and, therefore, improving the efficiency of biomass deconstruction [87,88]. The modularity of natural cellulosomes allowed the engineering of these proteins as platforms for coupling enzymes related to the production of biofuels and value-added chemicals from cellulosic biomass [89,90,91].

Integrating “accessory” enzymes to design cellulolytic cellulosomes resulted in the improvement of global enzymatic activity and biomass conversion. For example, Arfi et al. constructed a series of dockerin-fused lytic polysaccharide monooxygenases (LPMOs) and attached them to cohesin modules in designed cellulosomes together with an endo- and an exo-cellulase, which resulted in an increased release of soluble sugars from cellulose [90]. Similarly, integration of an expansin-like protein into natural and designed cellulosomes enhanced cellulose degradation by acting in synergy with the cellulosomal cellulases [89]. In another study, a laccase-like enzyme from *Thermobifida fusca* was fused to a dockerin–xylanase chimera and incorporated into a cellulosome already containing two cellulases and another xylanase. This resulted in a twofold increase in the amount of reducing sugars released from wheat straw when compared with the complex lacking the laccase [91].

Additionally, protein scaffolding was used in the creation of new complex designer structures [92,93], enabling the amplification of the number of enzymes attached into designed cellulosomes. For example, Stern et al. constructed an adaptor scaffoldin possessing three cohesins for integration of two endoglucanases and one exoglucanase from *T. fusca*, a CBM for targeting to the cellulosic substrate, and a dockerin for interaction with a hexavalent scaffoldin, designed to connect four xylanases and an additional endoglucanase also from *T. fusca*. As a result, they showed an increase in cellulose degradation in untreated lignocellulosic substrates [94]. By using an unusual and elegant approach, a double-stranded DNA (dsDNA) scaffoldin was constructed to connect several cellulases, simulating natural cellulosomes. The novel artificial cellulosome—DNA-(EG)_n_ conjugates—containing several units of endoglucanase Cel5A from *T. fusca* conjugated onto the DNA scaffold, showed a 5.7-fold enhancement of enzymatic saccharification [95]. 

Also, cellulosome-inspired complexes were proposed for increasing enzymatic efficiencies [96,97]. For instance, Kim et al. constructed artificial cellulosomes by nanoclustering. For this purpose, the catalytic domain of endoglucanase CelD from *Clostridium thermocellum* and different CBMs were biotinylated and bound to streptavidin–cadmium selenide nanoparticles as a scaffold. They showed that the artificial cellulosome built on nanoparticle scaffolds was capable of degrading cellulose [98]. 

##### Engineering Multifunctional Enzymes: One Enzyme with Multiple Activities

Creating artificial multiple domain enzymes has some advantages when compared with single-activity proteins. Two or more fused-catalytic domains can display increased catalytic activities, higher stabilities, and competitive production costs, as well as aid the straightforward substrate–product channeling due to the proximity of the enzymes [99,100,101]. Biomass-converting enzymes are relevant targets for the construction of multifunctional enzymes, which is reflected by the number of recent patents [102,103,104,105] and publications focused on the development and applications of such chimeric enzymes. In a recent study, Taylor et al. conducted a combinatorial permutation of the CBM, linker, and catalytic domains from two GH7 cellobiohydrolases, *Tr*Cel7A from *T. reesei* and *Pf*Cel7A from *Penicillium funiculosum*, and compared the performance of the subdomain-swapped chimeras. PfCel7A exhibited 60% greater performance on biomass than TrCel7A and swapping the CBM, or the CBM and linker of *Tr*Cel7A for that of *Pf*Cel7A improved *Tr*Cel7A performance by about 33% and 45%, respectively [106]. 

In another recent study, two bifunctional chimeras (Cel5A–XylT and XylT–Cel5A) were generated from genes encoding the heat-active endoglucanase (Cel5A) and endoxylanase (XylT) from *Fervidobacterium gondwanense,* isolated from hot thermal springs. The chimeras were constructed by end-to-end fusion and connected by a linker sequence (DKTKYTAS) of esterase EstO from *Pseudoaltermonas arctica*. Both chimeras showed increased specific activity on β-glucan and a significant improvement on specific activity toward beechwood xylan by 4.3- and 4.5-fold for Cel5A–XylT and XylT–Cel5A, respectively, when compared to parental XylT [107]. In an interesting work, Ribeiro et al. used rational design to engineer two bifunctional chimeras combining (1) a laccase and a xylanase from *B. subtilis*, and (2) swapping the parental xylanase by a previously engineered thermostable variant [108]. The bifunctional enzymes were constructed by inserting the xylanase into a surface loop of the laccase, resulting in a central region composed by either the xylanase or thermostable xylanase flanked by the N-terminal and C-terminal regions of laccase. Both chimeras presented increased catalytic efficiencies (*k*cat/*K*_m_) for laccase by around twofold when compared with the parental laccase [108].

In other examples of engineered multifunctional enzymes, GH11 xylanase (XynA) from *B. subtilis* was inserted either randomly [36] or by semi-rational design [35] into a xylose-binding protein (XBP) from *E. coli*, generating XynA–XBP libraries. After several steps of screening, chimeric variants were found with a novel allosteric behavior, in which xylanase activity became positively modulated by xylose. These chimeras also showed higher thermostability than the parental XynA.

#### 2.2.2. Engineering Protein Stability 

Protein stability could be considered as the ability of a protein to maintain its structure and function in a particular environment [109] or as the resistance of a protein to unfolding [110]. As stated by Deller et al., “protein stability means many different things to many different scientists” [109]. However, what is certain is that the viability of an enzymatic process is highly dependent on protein stability.

Industrial lignocellulose conversion requires pretreatment of biomass in order to make cellulose and hemicellulose available for saccharification. This pretreatment can be achieved in many ways, which mainly include acid or alkali treatment, liquid hot water, steam explosion, and, more recently, ionic liquids [111]. It implies that hydrolytic enzymes which can cope with heat, extreme pH, or ionic liquids are more compatible with pretreatments and, therefore, desired for the saccharification process. 

##### Engineering Thermostability

Due to recalcitrance of lignocellulose, pretreatment steps are essential to improve the accessible surface area for microorganisms and enzymes to work. Several pretreatment methods, such as conventional heating (high heat and pressure), microwave, steam explosion, hot combined liquid/vapor, and thermophilic biological pretreatment, are done at high temperatures [112]. In addition, high temperature is also needed to solubilize lignin and hemicellulose in water [113]. Thus, thermostability is generally a desired characteristic for enzymes involved in the biomass conversion process. For many lignocellulose-hydrolyzing enzymes, different protein engineering approaches were used for thermostability optimization per se or achieved it while searching for improved activity. Recent work on protein thermostability engineering in these enzymes is summarized in Table 2. In this endeavor, the search for thermostable *T. reesei* cellulases is of particular interest as they are the most widely used enzymes for lignocellulose conversion. *T. reesei* is a mesophilic organism and, therefore, its cellulases are only moderately tolerant to temperatures above 50 °C [114]; thus, considerable efforts were made in the last few years to enhance their thermostability.

*T. reesei* Cel6A and Cel7A are cellobiohydrolases that belong to family GH6 and GH7 of glycoside hydrolases, respectively [115]. They are the major components of *T. reesei* cellulolytic enzymes, representing Cel7A and Cel6A 55% and 18% of the total cellulase content, respectively [115]. Therefore, these cellobiohydrolases are great candidates for thermostability improvement. Smith et al. generated thermostable Cel7A variants from *Hypocrea jecorina* (anamorph *T. reesei*) by noncontiguous structured-guided SCHEMA recombination [65]. This methodology is based on the identification of structural blocks among homologous proteins that can be shuffled in order to generate chimeras with novel behaviors, such as improved thermostability [116]. By swapping structural blocks of Cel7A from *H. jecorina* and two thermostable homologs, the authors identified chimeras with improved thermostability related to 23 different mutations. Later, they used this information to construct 23 single-mutant variants of *H. jecorina* Cel7A by site-directed mutagenesis. Mutant F362M displayed increased stability by 3 °C (T_50_) and increased activity by ~3-fold at 49 °C, compared to parental *H. jecorina* Cel7A [65]. Lantz et al. reported using structure and sequence analysis to identify Cel7A sites potentially involved in stability, and used saturation mutagenesis to obtain Cel7A variants with improved stability. As the enzyme is patented, no further details are mentioned [117]. Later, Goedegebuur et al. selected some of those variants in order to improve the thermal stability of Cel7A through further steps of directed evolution. Combining 18 mutations, the authors obtained the variant FCA398 which exhibited a 10.4 °C increase in Tm, and a 44-, 34-, and nine-fold enhanced half-life at 62 °C, 66 °C, and 69 °C, respectively. Variant FCA398 retained relatively high activity even at 75 °C [66].

Heinzelman et al. used structure-guided contiguous SCHEMA recombination for the stabilization of *H. jecorina* Cel6A by incorporating stabilizing blocks from *Hypocrea insolens* and *Chaetomium thermophilum*. A highly thermostable chimera, HJPlus, was obtained which hydrolyzed cellulose at temperatures 7–15 °C higher than the parental enzymes and also outcompeted them in long-time activity (in the case of parental *H. jecorina*, by a factor of 1.7) [67]. Later, they used random mutagenesis and directed evolution for further stabilization of HJPlus. Three variants, 1G6, 2B3, and 3C6P, were obtained which exhibited a 1.6-, 4.2-, and 32-fold improvement in half-lives, respectively. Furthermore, T_50_ for the three variants increased by 1.3 °C, 3.8 °C, and 8.2 °C, respectively. The authors also proved that most of the identified mutations which improved thermostability in HJPlus had the same stabilizing effect when performed in Cel6A from *H. jecorina* [68]. 

*T. reesei* Cel7B and Cel5A are endoglucanases that represent approximately 9% and 8% of the total cellulase content, respectively [115]. Therefore, they are also interesting candidates for the improvement of thermostability. Chokhawala et al. used structure-guided evolution to engineer Cel7B of *T. reesei* for increased thermostability. Using a B-factor-guided approach, the authors identified 20 amino acids putatively involved in protein flexibility and subjected them to site-directed mutagenesis. One variant, G230A/D113S/D115T, resulted in a 3 °C increase in Tm (68 °C vs. 65 °C), ~4-fold enhancement in specific activity, and ~2-fold improved half-life at 60 °C when compared to parental Cel7B [69]. Bayram et al. engineered Cel7B from *T. reesei* by site-directed mutagenesis of amino acids putatively involved in thermostability, based on *in silico* predictions. One mutant, Q274V, increased its optimal temperature by ~10 °C with respect to parental Cel7B. Q274V was also more stable, maintaining ~80% and ~40% of its activity when incubated for 8 h at either 45 °C or 65 °C, respectively, while the parental Cel7B maintained ~40% and ~20% of its activity in these conditions [70]. Zhang et al. also used site-directed mutagenesis for improving the thermostability of *T. reesei* Cel7B. By an *in silico* approach, the authors identified “weak spots”, which are regions putatively involved in the initiation of partial unfolding, and introduced disulfide bonds to improve thermostability. All 13 mutants exhibited increased thermostability, with the best variant featuring a combination of mutations, G4C−F71C/N160C−G183C/S168T, which increased its T_50_ by 8.2 °C when compared to parental Cel7B. This mutant showed a ∼10 °C increase in optimal temperature when Avicel and filter paper (FP) were used as substrates. Interestingly, at 50 °C, the mutant displayed ∼1.3- and ∼2.5-fold increased activity in Avicel and FP, respectively, in comparison to parental Cel7B [71]. 

Akbarzadeh et al. used site-directed mutagenesis to eliminate two disulfide bonds of Cel5A of *T. reesei* in order to assess if their absence correlated with increased thermostability, as expected by structural comparison with thermophilic Cel5A from *Thermoascus aurantiacus*. When compared to the parental Cel5A, the thermal stability of variant C99V increased 2.4-fold at 80 °C, 2.01-fold at 70 °C, and 1.8-fold at 60 °C, while, for variant C323H, it increased 2.34-fold, 1.81-fold, and 1.6-fold at 80 °C, 70 °C, and 60 °C, respectively [62]. Trudeau et al. used site-directed mutagenesis for improving the thermostability of *H. jecorina* Cel5A by the combination of 16 previous mutations that proved to thermostabilize Cel5A. The resulting variant, OptCel5A, displayed an optimal temperature of 81 °C, which is 17 °C higher than that from parental Cel5A. In a period of 60 h, OptCel5A at 70 °C released ~1.5-fold more reducing equivalent from Avicel than parental Cel5A at 60 °C, which is its optimal temperature. Later, the authors assessed the synergism exerted by the combination of previously engineered Cel7A and Cel6A, together with OptCel5A, and compared it with the synergistic effect of the combination of parental Cel5A, Cel6A, and Cel7A. At 70 °C, the optimized engineered mixture, T-PRIMED, released three-, 1.8-, and 2.5-fold more reducing equivalents from Avicel, milled corn stover, and dilute acid-treated rice straw, respectively, when compared to the cocktail containing all parental enzymes at 60 °C [72].

All these recent works dedicated to *T. reesei* cellulase thermostability improvement, together with all the examples detailed in Table 2, demonstrate that protein engineering is an excellent tool for this endeavor, and that there is still much work to do in this regard. 

##### Engineering Ionic Liquid Stable Variants

Ionic liquids (ILs) are basically salts in the liquid state composed of an anionic and a cationic part [118]. ILs are considered a promising class of solvents able to dissolve the lignin–cellulose complex. Consequently, ILs were used as an alternative pretreatment under mild conditions. In order to decrease the cost to biomass conversion, IL pretreatment and saccharification may be performed simultaneously. However, in the presence of even low concentrations of ILs, most of the available enzymes are inactivated [119]. Therefore, the stability of hydrolytic enzymes in ILs is the most recent challenge for the development of a cost-effective coupling between pretreatment and saccharification. Extensive research in the field is currently dedicated to the search of IL-stable cellulases, and more recently to protein engineering for the development of IL-stable variants [119,120]. In this regard, Wolski et al. used DNA shuffling to evolve enzyme variants of *Talaromyces emersonii* Cel7A (TeCel7A) to be more active and stable in ILs [121]. Previously, they determined that *T. reesei* cellulases remain active in 40% (*w*/*w*) 1,3-dimethylimidazolium dimethyl-phosphate (Mmim DMP) [122] and, therefore, they used this IL for improvement of TeCel7A. They found two variants, 2K15 and 1M10, which, in the presence of 43% (*w*/*w*) Mmim DMP, produced more glucose than the parental TeCel7A when using Avicel pretreated with 1-ethyl-3-methylimidazolium acetate as substrate. The 2K15 and 1M10 variants also outcompeted *T. reesei* Cel7A, which was mostly inactive under these conditions [121]. Pottkamper et al. used sequence saturation mutagenesis for the search of variants of cellulase CelA_10_ with better stability toward IL. CelA_10_, which belongs to the GH5 family, was isolated in a function-driven metagenomic screening. Two variants, CelA_10M6_ and CelA_10M7_, showed fivefold increased activity in the presence of 30% (*v/v*) 1-butyl-1-methylpyrrolidinium trifluoromethanesulfonate when compared to parental CelA_10_ [123]_._ Chen et al. used error-prone PCR random mutagenesis for the search of variants of *Thermotoga maritima* cellulase Cel5A with higher activity on 1-ethyl-3-methylimidazolium acetate-pretreated switchgrass (IL-S). Variants N236D and H138R showed 30% and 22% increases in specific activity toward IL-S, respectively [124]. 

Identification of essential residues involved in IL tolerance of hydrolytic enzymes is crucial for the viability of the one-step pretreatment–saccharification process. Coupling IL pretreatment and saccharification would enable a cost-effective approach for second-generation biofuel and maybe in the future will allow for consolidated bioprocessing where pretreatment, saccharification, and fermentation take place in one reactor. 

#### 2.2.3. Engineering Functional Expression and Cellular Localization 

Efficient conversion of biomass relies heavily on the enzymes being able to access their substrates [125]. This implies that extracellular localization and/or membrane anchoring of lignocellulolytic enzymes is required. Both approaches, in addition to improving biomass degradation, may also allow the much-desired consolidated bioprocessing (CBP), which is a promising approach for the optimization of processes such as ethanol production [125,126]. In CBP, cellulose hydrolysis, saccharification, and fermentation occur simultaneously. 

**Figure 3:**
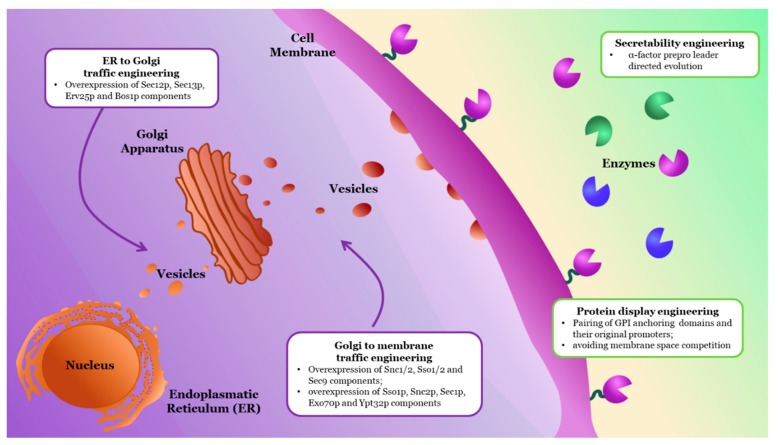
Strategies for the improvement of enzyme functional expression in yeasts. This figure illustrates the approaches discussed in further detail in the text. Typical targets for vesicle traffic engineering are listed within the purple (intracellular) area, whereas the green (extracellular) region shows the strategies employed to improve enzyme display and secretion.

Several strategies were developed for the improvement of fermentative strains in order to achieve the CBP, by genetically engineering them to express heterologous cellulolytic enzymes and either secreting them onto the extracellular medium or displaying them on their cell wall (Figure 3). 

A well-known strategy for improving secretion of a recombinant protein is by attaching a signal or leader peptide to the target protein, which will direct the processing and transport of the heterologous protein to its extracellular destination [127]. For instance, Camarero et al. improved the secretion of a *Pycnoporus cinnabarinus* high-redox laccase in *Saccharomyces cerevisiae* by switching its original signal peptide to the α-factor prepro-leader sequence, recognized by yeasts, and subjecting the protein fusion to rounds of directed evolution and selection, obtaining remarkable secretion increases of up to 40-fold [128]. 

In a follow-up study, both native and different evolved α-factor prepro-leader sequences were used as the starting point for generating laccase mutants with different redox potentials [129]. This work showed that using already evolved leader sequences for directed evolution could improve the functional expression of laccases better than using the native *S. cerevisiae* prepro-leader, regardless of their redox potential, and to which laccase the leader sequence was initially attached before directed evolution. Another study used Camarero’s evolved *S. cerevisiae* α-factor prepro-leader to successfully direct secretion of a laccase from the filamentous fungus *Coriolopsis gallica* in the yeast *Pichia pastoris* [130], showing that this approach might develop more universal signal peptides allowing heterologous protein expression in different hosts in the future.

Engineering components related to vesicle trafficking is another approach to improve the expression of heterologous proteins and was also explored as a novel way to enhance cellulolytic efficiency (Figure 3). For instance, Van Zyl et al. improved *S. cerevisiae* secretion of a cellobiohydrolase from *Talaromyces emersonii* (Cel7A) and a β-glucosidase from *Saccharomycopsis fibuligera* (Cel3A) by overexpressing specific Soluble N-ethylmaleimide-sensitive factor Attachment Receptor (SNAREs) genes, which encode small membrane proteins that coordinate intracellular protein trafficking from Golgi to the cell membrane [131]. The simultaneous over-expression of differential combinations of *Snc1/2*, *Sso1/2*, and *Sec9* genes, components of the exocytic SNARE complex, yielded a maximum increase of ~52% and ~49% in the secretion of Cel7A and Cel3A, respectively. It is worth noting that overexpression of different SNAREs did not have the same effect in both enzymes. For example, Cel3A was better secreted when Sso1 was overexpressed, while, for Cel7A, this was achieved with Snc1 overexpression.

Tang et al. assessed the effect of engineering vesicle trafficking components in extracellular secretion and surface display of an endoglucanase (CelA) and a β-glucosidase (BGL1) [132]. Overexpression of vesicle trafficking components Sec12p, Sec13p, Erv25p, and Bos1p, involved in trafficking from the endoplasmic reticulum to Golgi, improved the secretion of both enzymes in different levels, showing a protein-specific effect of these transport pathways similar to that observed by van Zyl et al. [131]. However, even though overexpression of protein transport components from Golgi to the plasma membrane was more effective for BGL1 secretion, it was suggested for the first time that vesicle engineering could be a viable approach not only for secretion but also for cell-wall surface display of heterologous enzymes, given that they successfully obtained CelA and BGL1 fused to surface-anchored protein α-agglutinin.

Cell-surface display of enzymes is an exciting approach for alleviating the cost of fermentation, given that the attached enzymes remain viable as long as the cells are thriving in the environment. Additionally, cells can be re-used from batch to batch, lowering the need for both inoculum propagation and enzyme addition in industrial processes [133,134]. Native membrane proteins such as α-agglutinin rely on GPI (glycosylphosphatidylinositol)-anchoring domains for efficient attachment to the cell surface, and these domains can be used to fuse heterologous proteins to the yeast surface [135].

Inokuma et al. assessed the effect that coupling the GPI promoter and anchoring regions of two membrane proteins (Sed1 ─Secreted protein containing EGF repeats and Discoidin/F5/8 complement domains─ and α-agglutinin) had on β-glucosidase (BGL1) or endoglucanase (EGII) activity in *S. cerevisiae* [136]. Interestingly, those cassettes with paired anchor–promoter showed higher enzymatic activity on the cell-wall surface when compared to cassettes with two unrelated genetic elements. These results suggested a novel synergistic effect caused by using a membrane protein GPI-anchoring domain and its original promoter sequence for the wall-surface display of heterologous enzymes. Interestingly, subsequent work by Bamba et al. studied the effect of the Sed1–BGL1 cassette in a *S. cerevisiae* strain with SED1 protein disrupted. The SED1 mutant displayed higher cellulolytic activity than the wild-type strain, suggesting a competition for membrane space between the native SED1 and the cell-wall associated cellulolytic enzymes harboring the Sed1-anchoring domain [137]. These two works suggest that the combination of promoter and anchoring region, together with disruption of native cell-wall-associated proteins, might be a powerful approach for the improvement of anchoring efficiency and activity of heterologous enzymes bound to the cell wall.

Even though this section focused on eukaryotic models, it is worth noting that several approaches were also taken in prokaryotic hosts to pursue a more efficient protein secretion and display, given that purification of such enzymes is often troublesome due to the typical formation of inclusion bodies in these systems [126]. Works by Yildirim et al., in which a recombinant endoglucanase was efficiently engineered for periplasmic and extracellular targeting by a native secretion signal of *E. coli* [138], and Huang et al., who showed the advantages of using protease-deficient strains of *B. subtilis* for cell-surface display of heterologous endoglucanase [139], gave insights for overcoming current limitations of prokaryotic systems. 

Overall, the several approaches presented in this section show the abundance of methodologies available for improving the functional expression of heterologous proteins, allowing the design of more sophisticated strategies for optimized biotechnological ventures.

#### 2.2.4. Protein Engineering Guided by Deep Mutational Scanning

Systems biology helped to develop large-scale and holistic approaches to improve our understanding of complex biological systems, while synthetic biology provided us with a higher capacity to create genetic circuits and pathways through synthesis. Deep mutational scanning was established as a technique combining the advances in nucleotide synthesis and sequencing, to explore the mutational landscape in protein sequences [140]. 

This approach consists of the functional screening and deep sequencing of an (ideally) unbiased library of protein mutational variants. Selective pressure for the desired function is applied to the constructed library. Clones that were enriched by this pressure are then identified by deep sequencing techniques. The only requirement of the screening methodology is that the nucleotide sequence that originated the protein can be obtained at the end of the screening. A detailed protocol for the implementation of deep mutational scanning and a thorough perspective article are available for the reader [141,142]. 

Mutational scanning experiments were used to guide improvements in a variety of proteins characteristics, like enzyme stability [143], solubility [144], and membrane interaction [145], in proteins that present a variety of different functions and structural motifs. Although few works were used to guide improvements in biomass-converting enzymes, the versatility of the technique and its potential to provide insights into an extensive library of enzyme mutants makes this technique suitable for future applications. This tool for protein analysis has great potential to uncover central protein regions that would not have been identified otherwise. Even though deep mutational scanning increased our comprehension of protein fitness, the technique was, until recently, limited to enzymes that can be directly related to population growth.

Romero et al. described a robust methodology, combining what is known as drop-based microfluidics with deep sequencing techniques, to analyze a library of 3083 point-mutation variants of a glycosidase from *Streptomyces* sp. [146]. In their work, a custom-built nanodrop sorter was used to select active clones of the glycosidase using a fluorogenic substrate which was compartmentalized in a droplet with cell extract. Those droplets were then sorted according to enzyme activity and subjected to Illumina sequencing. After validating the approach, a high-temperature enriching assay was performed within the library to identify mutations related to thermal stability. One of those point mutations increased *T_50_* of the enzyme by 5.3 °C, exemplifying how the technique can be used to generate catalysts suitable for industrial settings.

Klesmith and coworkers described exciting insights for a levoglucosan kinase in the light of deep mutational scanning. One of their approaches, termed FluxScan, achieved a 24-fold improvement in the enzyme activity [147]. Interestingly, point mutations that improved activity were scattered throughout the protein, and one of the clones could not sustain enzyme activity when expressed by a strong promoter, showing that the genetic context can be a challenging factor when screening for improved biocatalytic activity.

Although a primarily descriptive method, deep mutational scanning, when coupled with robust selection systems, provides a comprehensive fitness landscape snapshot that can be used to uncover regions that are less prone to collapse following protein engineering, and to find mutations that confer desirable properties.

## 3. Concluding Remarks and Future Perspectives 

Designing state-of-the-art enzymes to act in bioprocesses is gradually becoming a reality. Here, we briefly presented several genetic approaches for protein engineering to overcome barriers for protein adaptation during the biomass conversion process. We focused on the improvement of protein activity, stability, functional expression, and cellular localization. A broad set of molecular and computational tools emerged in the last 10 years. These tools allowed creating “smarter” libraries and more efficient screening methods. Thus, when choosing the features of the protein to be engineered, researchers have several options to decide with regard to the genetic diversification strategy, as well as the more suitable screening or selection technology.

Moreover, it is relevant to highlight that, over the years, the information generated by directed evolution was used to increase the knowledge about the protein fitness landscape, allowing us to understand better how a particular protein evolved. The increasing availability of this information, enhanced by deep sequencing and structural data, and coupled with advancements in computational prediction, led to a trend toward semi-rational and rational approaches. These focused approaches could reduce the time for protein improvement from years to months. Computational and automated designing of enzymes with accurate protein scaffolds is still hard to reach today. A remarkable work recently published showed the design of 21 active xylanases GH10 through an automated combinatorial backbone assembly method [148]. The method assembles new backbone combinations using Rosetta and PROSS computational tools to optimize the amino-acid sequence. More ambitiously, tailor-made proteins may be created by de novo design (with sequences unrelated to those in nature). The de novo protein design is outside of the scope of this review; however, an excellent article by Huang, Boyken, and Baker may be consulted [149]. 

Experimentally, the in-progress advances in gene synthesis, deep sequencing, continuous evolution, or ultrahigh-throughput screening methods are already generating innovative and exciting discoveries of novel biocatalysts. These novel enzymes, in addition to being more active or adapted to the bioprocesses, should also be cost-effective for industrial application. Moreover, the research for more efficient biomass conversion by microbial enzymes also requires the ability to engineer enzymes in a genome context. Currently, the clustered regularly interspaced short palindromic repeat/associated protein 9 (CRISPR/Cas9) system is the most promising tool for genome editing [150]. CRISPR/Cas9-based tools for directed evolution [151] and rational design [152] could become an attractive option for fast and efficient protein engineering of enzymes on the chromosomes of industrial strains. It is only the beginning!

## Figures and Tables

**Table 1:** Analysis of some codons used for saturation mutagenesis.

Degenerate Codon ^a^	Number of Codons	Number of Amino Acids	Number of Stop Codons	Encoded Amino Acid	Library Size for 2 Positions ^b^	Library Size for 3 Positions ^b^
NNN	64	20	3	All	995	25585
NNK	20	20	1	All	875	21051
NNS	32	20	1	All	875	21051
DBK	18	12	0	ARCGILMFSTWV	279	3812
NDT	12	12	0	RNDCGHILFSYV	215	2587
NRT	8	8	0	RNDCGHSY	95	766
NAN	16	7	2	YHNQKDE	95	766
NTN	16	5	0	MFLIV	62	409
NCN	16	4	0	SPTA	23	95
RST	4	4	0	AGST	23	95

^a^ IUPAC terminology [47] **N** = A/C/G/T; **K** = G/T; **S** = C/G; **D** = A/G/T; **B** = C/G/T; **R** = A/G. ^b^ To ensure a 0.95 probability of discovering at least one of the top two variants. The library sizes were calculated using an online tool [48] that can be found at the following website: http://trachel-srv.cs.haifa.ac.il/rachel/toplib/.

**Table 2:** Recent progress in engineered biomass-converting enzymes.

Enzyme	Organism Source	Methods ^a^	Variant	Improved Characteristic ^b^			Refs
				Activity	Thermostability	pH	
**endo-β-1,4-glucanase** **(GH12)**	*Streptomyces* sp. G12	epPCR	1) epCS_2 (G145D/ N207K) 2) epCS_1 (G263C/ R307H) 3) epCS_4 (T67N/ D142E/ S218N/ V242D/ D330E)	↑ ~1.4-fold, ~1.4-fold, ~1.7-fold in activity at 60 °C for epCS_2, epCS_1 and epCS_4, respectively, in AZO-CMC	34% remaining activity after 72 h at 60 °C for epCS_1 (17% for WT)	epCS_4 activity ↑ ~2 fold at pH 5 and ~2.6 fold at pH 6	[54]
**GH5 cellulase Cel5**	*Gloeophyllum trabeum* CBS 900.73	SDM	1) N233A 2) N233G	↑ k_cat_/K_M_: 45% and 52% for N233A and N233G, respectively, using CMC as substrate	NSI	N233G retained ~75% activity after 1h at 37 °C in pH 2 (~30% for WT)	[55]
**β-mannanase**	*Bacillus* sp. MK-2	epPCR	1) Q112R 2) L211I 3) K291E	↑ k_cat_/K_M_: 2.8-, 1.7- and 4.2-fold for Q112R, L211I, and K291E, respectively, using konjac glucomannan as substrate	NSI	ND	[56]
**CelA GH9 and CelA GH48**	*Caldicellulosiruptor bescii*	rational design	1) D5-GH48 2) D5-GH9	D5-GH48 30%↑ activity in Avicel D5-GH9 82%↑activity in CMC	ND	ND	[57]
**XynB xylanase**	*Aspergillus niger* ATCC1015	SDM	1) T43E 2) S41N/T43E	↑ k_cat_/K_M_: 20% and 65% for T43E and S41N/T43E, respectively	S41N/T43E: 60% and 35% remaining activity after 60 min and 120 min at 60 °C, respectively (~12% for WT at both times)	ND	[58]
**XynCDBFV xylanase**	*Neocallimastix patriciarum*	SDM	1) W125F 2) F163W 3) W125F/F163W	↑ enzyme activity: 10% for single mutants and 20% for double mutant using xylan beechwood as substrate	W125F/F163W slightly more residual activity (~6-8%) at 80 °C and 90 °C for 120 and 30 min, respectively.	ND	[59]
**β-glucosidase GH3 (BGL1)**	*A. niger*	directed evolution	1) Y305C 2) Q140L	Y305C reduced transglucosylation activity Q140L reduced the inhibitory effect of glucose	ND	ND	[60]
**Cel5A**	*Bacillus agaradherans*	SDM	1) N141L 2) A137Y 3) I102A/A137Y	3-, 1.5-, and 3.4-fold ↑ activity for N141L, A137Y and I102A/A137Y, respectively	ND	ND	[61]
**Glucanase II (Cel5A)**	*Trichoderma reesei*	SDM	1) C99V 2) C323H	↑ k_cat_/K_M_ 1.87-fold and 1.3-fold for C99V and C323H, respectively, using CMC as substrate	↑*T_1/2_* 2.4-fold (80 °C), 2.01-fold (70 °C), 1.8-fold (60 °C) for C99V ↑*T_1/2_* 2.34-fold (80 °C), 1.81 -fold (70 °C), 1.6-fold (60 °C) for C232H	ND	[62]
**GH5 Cel5E**	*Clostridium thermocellum*	rational engineering using overlapping PCR	1) N94W 2) N94F 3) E133F 4) N94A	N94W, N94F, E133F, and N94A mutants showed 1.92-, 1.29-, 1.1-, and 1.15-fold ↑ CMCase activity and 1.46-, 1.29-, 1.11- and 1.12-fold ↑β-glucanase activity on barley β-glucan	N94W, N94F, E133F, and N94A retained 92%, 91%, 93%, and 90% of residual CMCase activity (WT ~86%) and 91%, 89%, 91%, and 88% of residual β-glucanase activity (WT 82%), during 4 h at 62 °C	N94W ~2-fold and 1.5-fold ↑activity at pH 6–8 in CMC and barley β-glucan, respectively	[63]
**cellulase cocktail from *T. reesei***	*T. reesei*	succinylation	change enzyme surface charges	2-fold ↑ in cellulose conversion in 15% (*v/v*) 1-butyl-3-methylimidazolium chloride and further reduction in apparent K_M_ of the enzyme cocktail for Avicel by 2.7-fold due to reducing lignin inhibition	ND	ND	
**family 11 alkaline xylanase Xyn11A-LC**	*Bacillus* sp. SN5	epPCR	1) V116A/ E135V 2) E135V 3) E135R	1.1-, 1.5-, and 1.3-fold ↑ in catalytic efficiencies for V116A/ E135V , E135V, and E135R, respectively	E135V and E135R, 73.1% and 77.8% remaining activity, respectively, at 60 °C for 30 min (33.5% for WT)	E135V ↑relative activity regarding the WT: 17.5% at pH 8.5, 18.9% at pH 9 and 14.3% at pH 10 E135V and E135R retain over 80% of activity in pH 4.5–10 (WT in pH 8–10)	[64]
**Cel7A**	*Hypocrea jecorina*	SCHEMA	F362M	↑ activity by ~3-fold at 90 min and 49 °C on MUL	↑ stability by 3 °C	ND	[65]
**Cel7A**	*H. jecorina*	directed evolution	FCA398 (S8P/T41I/N49S/A68T/N89D/S92T/S113N/S196T/P227L/D249K/T255P/S278P/E295K/T296P/T332Y/V403D/S411F/T462I)	↑ activity by ~2-fold at 70 h and 65 °C on PASC	↑Tm in 10.4 °C and ↑*T_1/2_* by 44-fold, 34-fold, and 9-fold at 62 °C, 66 °C, and 69 °C, respectively.	ND	[66]
**Cel6A**	*H. jecorina*	SCHEMA, random mutagenesis and directed evolution	1) HJPlus (not specify) 2) 1G6 (S317P respect to HJPlus) 3) 2B3 (Q277L/S317P respect to HJPlus) 4) 3C6P (S30F/V128A/M135L/Q277L/S317P/S406P/S413P respect to HJPlus)	↑in activity on Avicel after 2h: ~1.25-fold, ~1.8-fold, and ~6.5-fold at 60 °C, 65 °C, and 70 °C, respectively, for both HJPlus and 3C6P ~9-fold and ~15-fold at 75 °C, 8-fold and 28-fold at 80 °C for HJPlus and 3C6P, respectively ~16-fold at 85 °C for 3C6P	3.5-fold, 5.6-fold, 14.8-fold and 112-fold ↑ in T_1/2_ for HJPlus, 1G6, 2B3 and 3C6P, respectively. 6.7 °C, 8 °C, 10.5 °C and 14.9 °C ↑ in T_50_ for HJPlus, 1G6, 2B3 and 3C6P, respectively.	~3-fold and ~5-fold ↑activity at pH 7 and pH 8 for HJPlus	[67,68]
**Cel7B**	*T. reesei*	structure-guided evolution	G230A/D113S/D115T	↑ in specific activity after 15 h: ~4-fold at 50 °C on CMC ~1.4 and ~2-fold at 60 °C and 65 °C, respectively, on Avicel ~1.6 and ~3-fold at 60 °C and 65 °C, respectively, on MUC	↑Tm in 3 °C and ↑ T_1/2_ by ~2-fold at 60 °C	ND	[69]
**Cel7B**	*T. reesei*	SDM	Q274V	NSI	~10 °C ↑ in optimal temperature ~80% and ~40% remaining activity at 45 °C and 65 °C, respectively, for 8 h (WT ~40% and ~20%)	Retained 70% activity at pH 4 (WT 20%)	[70]
**Cel7B**	*T. reesei*	SDM to introduce disulfide bonds	G4C -F71C/N160C-G183C/S168T	↑ in specific activity ∼1.3-fold and ∼2.5-fold in Avicel and FP, respectively, after 24 h at 50 °C	8.2 °C ↑ in T_50_ and ∼10 °C ↑in optimal temperature	ND	[71]
**Cel5A**	*H. jecorina*	SDM	OptCel5A (F191V/T233V/V265T/S318P/D271Y/S79P/E53D/T57N/G293A/V101I/N155E/T80E/S133R/G239E/S309W/G189S)	↑~1.5-fold in activity on Avicel after 60h at 70 °C in comparison to the WT at 60 °C	17 °C ↑ in optimal temperature	ND	[72]
**β-glucosidase MeBglD2**	Isolated in a function-driven metagenomic screening	SDM	1)N59C/A295G 2)H8L/N59C/A295G	NSI	∼10 °C ↑in optimal temperature for both variants 7 °C and 9 °C ↑ in Tm for N59C/A295G and H8L/N59C/A295G, respectively. 50% remaining activity at 62 °C for 30 min for both variants (WT inactive already at 56 °C)	ND	[73]
**LPMO**	*Streptomyces coelicolor* LPMO10C	SDM	A143C-P183C/ S73C-A115C	ND	12 °C ↑ in Tm and 60% remaining activity after 2 h at 80 °C (WT 30%)	ND	[74]
**BglT**	*Bacillus terquilensis*	ISM	E46P/S43E/ H205P/S40E	↑ specific activity 64.4% after 10min at 40 °C on AZO- barley β-glucan and ∼1.5-fold ↑ in k_cat_/K_M_	∼20 °C ↑ in optimal temperature and 13.8 °C ↑ in Tm ↑T_1/2_ by 3.86-fold and 7.13-fold at 60 °C and 70 °C, respectively	Optimal pH shifted from pH 6.5 to pH 6.0 Retained 63.1% and 80.7% activity at pH 4.5 and pH 5.5, respectively (WT 7.9% and 59.9%)	[75]
**laccase**	*Bacillus* HR03	SDM	1) T415G 2) T415I 3) T418I	↑k_cat_/K_M_ 1.5-, 3.7-, and 1.5-fold on ABTS for T415G, T415I and T418I, respectively	↑T_1/2_ by 2-fold for T415I at 80 °C	ND	[76]
**xylanase**	*Orpinomyces* sp. PC-2	directed evolution guided by molecular dynamics	SM2 (V135A/A226and T and N-terminal region removed)	30,250-fold increase in k_cat_/K_M_ in beechwood xylan (2.9 × 10^10^ mL min^−1^ mg^−1^ vs. 8.0 × 10^6^ mL min^−1^)	↑T_1/2_ by 38-fold at 50 °C	Retained 45% activity at pH 3 (WT ~10%)	[77]
**xylanase XynAS9**	*Streptomyces* sp. strain S9	SDM	1)V81P/G82E 2)V81P/G82E/D185P/S186E	NSI	↑T_1/2_ by 16- and 14-fold at 65 °C, and 3.7- and 4.5-fold at 80 °C, for V81P/G82E and V81P/G82E/D185P/S186E, respectively 6.9 °C and 11.2 °C ↑in Tm for V81P/G82E and V81P/G82E/D185P/S186E, respectively 17 °C ↑ in optimal temperature for both variants	ND	[78]
**Family-11 xylanase**	*Paenibacillus campinasensis*	directed evolution and SDM	1) XynG1-1B43(V90R/P172H) 2) XynG1-1B43cc16(V90R/P172H/T84C-T182C/D16Y)	1.2- and 1.4-fold↑in k_cat_/K_M_ for XynG1-1B43 and XynG1-1B43cc16, respectively, on beechwood xylan	10 °C ↑ in optimal temperature for both variants 24.5% remaining activity at 90 °C for 120 min for XynG1-1B43cc16 (WT inactive)	~3.5-fold ↑activity at pH 11 for XynG1-1B43cc16.	[79]

^a^ SDM: Site-directed mutagenesis; epPCR (error-prone PCR); ISM: Iterative saturation mutagenesis ^b^ ND: not determined; NSI: no significant improvement regarding the wild type (WT); PASC: phosphoric acid-swollen cellulose; MUL: 4-methylumbelliferyl lactopyranoside; MUC: 4-methylumbelliferyl cellobiose; FP: filter paper, AZO-CMC: Carboxymethyl cellulose dyed with Remazolbrilliant Blue; CMC: Carboximethyl cellulose; AZO- barley β-glucan: barley β-glucan dyed with Remazolbrilliant Blue; ABTS: 2,2'-azino-bis(3-ethylbenzothiazoline-6-sulphonic acid)Using CBMs to Enhance Enzyme Activity.
